# Measuring the duration of kangaroo mother care for neonates: a scoping review

**DOI:** 10.1136/bmjopen-2023-079579

**Published:** 2025-01-22

**Authors:** Victor Spector Tumukunde, Eva Loucaides, Melissa M Medvedev, Moffat Nyirenda, Joy Lawn, Cally J Tann

**Affiliations:** 1 Faculty of Epidemiology and Population Health, London School of Hygiene and Tropical Medicine, London, UK; 2 Medical Research Council/Uganda Virus Research Institute and London School of Hygiene and Tropical Medicine Uganda Research Unit, Entebbe, Uganda; 3 Maternal, Adolescent, Reproductive, & Child Health Centre, London School of Hygiene and Tropical Medicine, London, UK; 4 Department of Pediatrics, University of California San Francisco, San Francisco, California, USA; 5 Department of Neonatal Medicine, University College London, London, UK

**Keywords:** NEONATOLOGY, PERINATOLOGY, PUBLIC HEALTH

## Abstract

**Abstract:**

**Objectives:**

Kangaroo mother care (KMC) is high impact for survival of low birth weight neonates, but there are few rigorous evaluations of duration required for impact. We conducted a scoping review of KMC duration measurement methods and assessed their validation.

**Design:**

Scoping review in accordance with Joanna Briggs Institute guidance for conducting scoping review.

**Data sources:**

MEDLINE, Embase, Cochrane Library, PsycINFO, African Index Medicus, Latin American and Caribbean Health Sciences Literature, ClinicalTrials.gov, International Clinical Trials Registry Platform, International Standard Randomised Controlled Trial Number Registry, Medrxiv and OpenGrey were searched through November 2022.

**Eligibility criteria for selecting studies:**

Publications with primary data on KMC duration were included. We excluded short procedural skin-to-skin care studies.

**Data extraction and synthesis:**

Selection and data abstraction were conducted by two independent reviewers. A data charting form based on the variables of interest was used to abstract data.

**Results:**

A total of 213 publications were included, of which 54 (25%) documented a method of measuring KMC duration. Only 20 publications (9%) provided a detailed description of the duration measurement method, and none reported validity. Most studies used caregiver reports (29, 54%) or healthcare worker observations (17, 31%). Other methods included independent observers and electronic monitoring devices.

**Conclusion:**

Only 9% of KMC studies reporting duration documented the measurement method applied, and no studies were found with documented validation of duration measurement methods. Accurate and comparable data on the dose response of KMC will require duration measurement methods to be validated against a gold standard such as an independent observer.

**STRENGTHS AND LIMITATIONS OF THIS STUDY**

- Unrestricted search across databases and grey literature reduced publication bias.
- Selection and data abstraction were conducted by two independent reviewers.
- Pilot testing and support from a clinical research specialist ensured precise data collection.
- Excluded five publications for which no English version was available.
- Did not review journal supplementary materials for the included publications.

## Introduction

 Globally, an estimated 2.3 million neonatal deaths occurred in 2022.^1^ More than 80% of neonatal deaths occur among those who are low birth weight (LBW, ≤2500 g), due to being born preterm, small-for-gestational age or both.^2^ Mortality risk is highest in low-income and middle-income countries (LMICs) due to gaps in neonatal care.^3^ Major mortality reductions could be achieved by improving facility-based care of small and sick neonates in these countries.^2 4 5^ Kangaroo mother care (KMC) as a component of this small and sick newborn care is associated with decreased mortality, sepsis, hypothermia, hypoglycaemia and length of hospital stay compared with conventional incubator care among clinically stable neonates.^6^,^8^ A WHO-led trial recently reported a 25% reduction in mortality within 28 days among neonates born weighing 1000–1799 g who received KMC immediately after birth, relative to those who received standard care with KMC after stabilisation.^9^ Based on these findings and additional evidence from a systematic review,^10^ WHO updated guidelines recommending KMC for all preterm or LBW neonates to be initiated as soon as possible after birth in the healthcare facility or at home and should be given for 8–24 hours per day.^11^

KMC is the care of preterm or LBW neonates in continuous and prolonged (8–24 hours per day, for as many hours as possible) skin-to-skin contact (SSC) recommended to be initiated immediately after birth with support for exclusive breastfeeding or breast-milk feeding.^12^ Duration of KMC is considered important in achieving beneficial health outcomes.^8 10 13 14^ However, previous research has suggested that continuous KMC for 24 hours a day may be difficult to achieve; for example, women may have complications or be post-caesarean section or find long hours challenging due to incompatibility with household activities or trying to sleep while continuing KMC.^15^ Policymakers and healthcare administrators should improve facility infrastructure and implement policies that encourage family support and involvement in KMC to improve duration.^11^

A higher duration of KMC in a given 24-hour period has been demonstrated using descriptive data and meta-analyses to be associated with lower mortality risk.^8 16^ Evidence also shows that some desired effects disappear when the KMC duration is 2 hours or less.^17^ However, the evidence base on the recommended frequency and duration of KMC for neonatal survival requires more rigorous evaluation.^9 15^ Dose-response studies could inform families and clinicians to optimise outcomes and be more efficient for inputs. Such studies require objective and accurate methods of measuring the duration of KMC.

The aim of this scoping review was therefore to explore available evidence on the methods used to measure KMC duration. Specific objectives were to (1) develop a framework for categorisation of measurement methods identified in the published and grey literature, (2) assess studies with KMC duration data to describe the measurement methods used and (3) describe any studies identified which validated duration measurement methods.

## Methodology

### Study design

We conducted a scoping review of the published and grey literature in accordance with established guidance for conducting a scoping review from the Joanna Briggs Institute.^18^ The review protocol was registered with Open Science Framework.^19^ Selection of relevant papers, screening and data charting were conducted by two independent reviewers (VST and EL) to minimise selection bias.

### Search strategy

We searched the MEDLINE, Embase, Cochrane Library, PsycINFO, African Index Medicus, Latin American and Caribbean Health Sciences Literature, ClinicalTrials.gov, International Clinical Trials Registry Platform and the International Standard Randomised Controlled Trial Number Registry. We also searched Medrxiv and OpenGrey libraries for relevant unpublished studies. We screened all references of relevant systematic reviews identified as well as the websites of the Kangaroo Foundation and the International Network of Kangaroo Care. WHO guidelines and Google Scholar were searched for relevant publications. Searches were first done 15 October 2020 last updated on 21 November 2022 with no language or date of publication limitations. Search terms were based on those relating to KMC and LBW/prematurity as well as KMC measurement/monitoring (online supplemental file appendix 1). The search was conducted with the assistance of a library clinical research specialist at the British Medical Association.

### Management of search results

The search results were exported as RSI files to the Mendeley reference management system (2009–2013, Mendeley). Duplication removal as well as title and abstract screening were done using Mendeley. The search results were shared with the second reviewer through a Mendeley group. Retrieved publications from the search were screened for suitability and relevance based on the information in the titles and abstracts. Initially, a randomly selected trial set of search results (10% of the total number) were screened for inclusion based on title and abstract information by both reviewers and, where necessary, clarifications/adjustments to the inclusion criteria were made, aiming for an agreement rate of >80%. A third reviewer was consulted in cases of disagreement (CJT). Articles were screened by two reviewers for inclusion and data charting. An initial pilot set was screened by both reviewers to assess agreement rates before sharing the analysis of the bulk of included articles.

### Eligibility criteria

Publications were included if they presented primary data on KMC among preterm or LBW newborns and referred to the duration of the skin-to-skin component of KMC or KMC monitoring/measurement. Publications referring to short procedural skin-to-skin care, such as delivery room routine skin-to-skin care and pain control procedures which did not fit in the definition of KMC,^12^ were excluded.

### Data abstraction

We generated a data charting form by identifying variables that would inform the objectives of the scoping review. Data points of interest included KMC duration, methods used to measure the duration and the validation of the method used. Detailed methodological description was defined as a study that explained the instruments used to document KMC duration measurement, the interval of the observations and how the total or daily KMC duration was calculated from the observations.

### Patient and public involvement

There was no patient or public involvement in this review.

## Results

**Figure 1:**
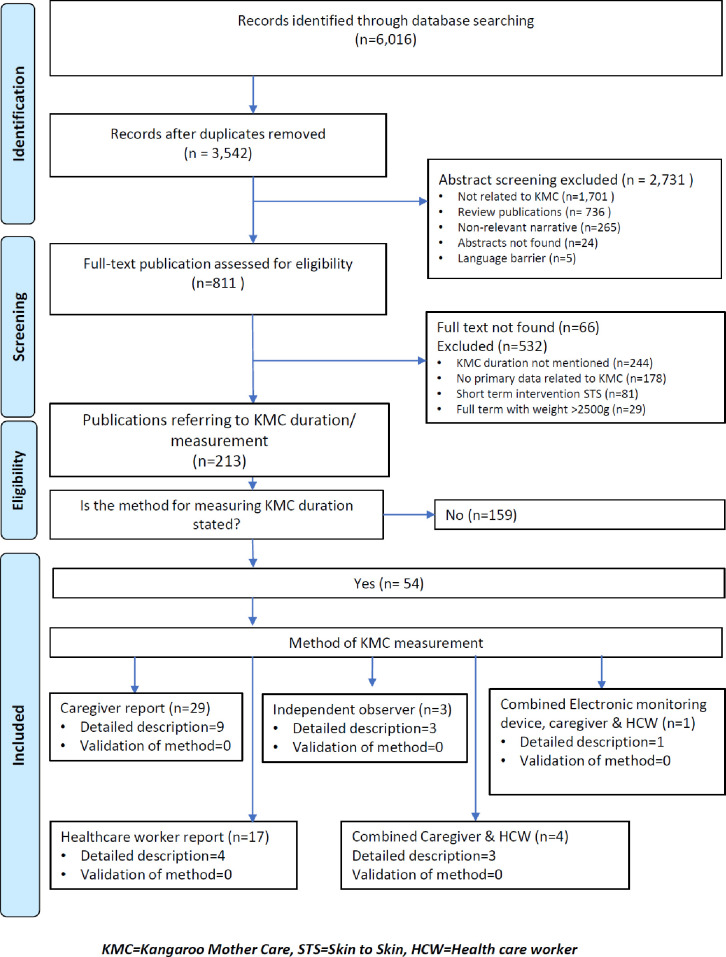
Preferred Reporting Items for Systematic Review and Meta-Analysis flow diagram of search results and study inclusion process.

Our search strategy identified 3542 publications, of which 213 presented primary data on KMC duration. Only 54 (25%) of 213 publications documented the method used to measure KMC duration (figure 1). Of the 213 publications, 139 (65%) were carried out in LMIC, 109 (51%) were clinical trials, 135 (63%) had a sample size of >50 participants and 102 (48%) reported on daily KMC duration of more than 2 hours.

### KMC duration measurement methods

Of the 54 publications that documented the methods used to measure KMC duration, four different methodological categories were identified: caregiver report, healthcare worker report, independent observation and electronic monitoring device. A method was identified as a healthcare worker report if a person involved in the routine care of study participants reported on KMC duration and as an independent observation if the person reporting on duration was not involved in the routine care of study participants. Some studies used more than one method, and caregiver reports were either self-reported through interviews or based on KMC charts/diaries.

**Figure 2:**
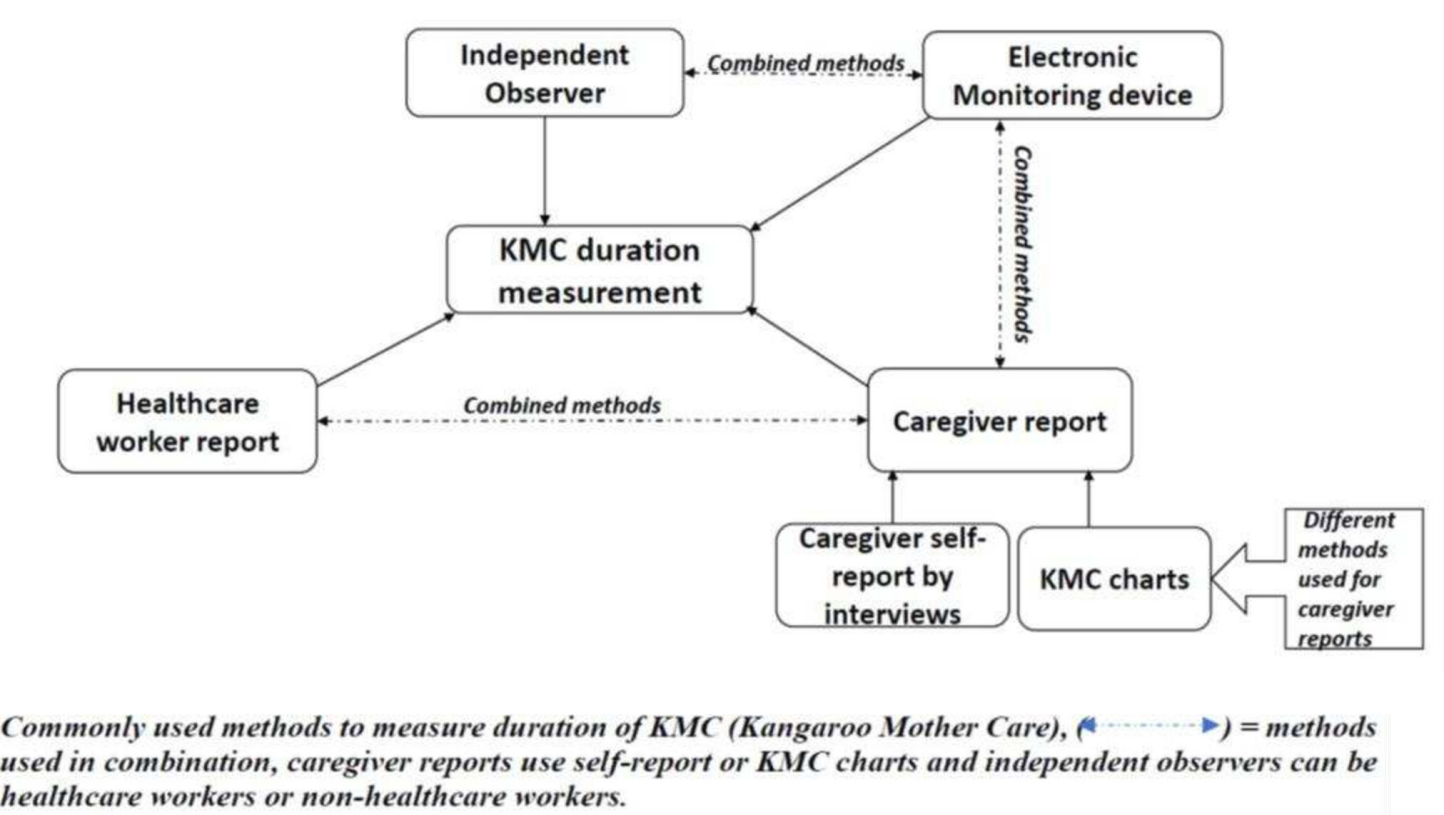
Proposed framework for categorisation of KMC measurement methods.

No existing framework for categorisation of the methods used to measure KMC duration was found in the reviewed publications, nor was any basis for the choice of the method used provided. Figure 2 illustrates our proposed framework for the categorisation of KMC measurement methods.

Of the 54 publications that documented the method used to measure KMC duration, 29 (54%) used caregiver reports and 17 (32%) used healthcare worker reports. Other methods included independent observation, a combination of healthcare worker and caregiver report, and a combination of electronic devices (wearable sensor for determining skin contact), healthcare worker and caregiver monitoring (figure 1).

### KMC duration measurement description

Nine (31%) of the 29 publications that used caregiver report described the method used to measure KMC duration. Seven of these nine publications used KMC charts/diaries to report the duration of SSC,^20^,^26^ while the remaining two mentioned self-report through interviews.^27 28^ Four publications^29^,^32^ used more than one method of measuring KMC duration. Of these, 3^29 31 32^ compared caregiver report with healthcare worker report and one^30^ used healthcare worker report and an electronic monitoring device to monitor skin-to-skin contact. In the latter study, the device was used in the home setting in combination with caregiver report to evaluate whether it could reliably capture the duration of KMC episodes.^30^

Three publications used independent observers^33^,^35^ who were not part of the healthcare team. No publication used video recording to monitor KMC duration although video was used to assess other aspects of SSC, such as mother-baby interaction.

Only 20 (9%) out of 213 publications^20^,^3032^ with primary data on KMC duration described in detail the measurement method used (figure 1), and this was in varying degrees of detail (online supplemental table 1). Of these 20 publications, 11 (55%) were from LMICs^2027 28 30 32^,^36 38 39^ and 9 (45%) were from high-income countries.^21^,^2629 31 40^ Nine (45%) out of the 20 publications used caregiver report (figure 1), of which 7 documented the tool used for monitoring KMC duration (charts)^20^,^26^ and 2 mentioned self-report (online supplemental table 1).^27 28^ The majority (64%) of the publications that used caregiver report were conducted in high-income countries,^21^,^26^ all of which documented the interval of observations. None of the publications gave a description of how the total or daily KMC duration was computed from the reports/observations (online supplemental table 1).

Three of the four publications that used healthcare worker report were conducted in LMICs.^36 38 39^ Only one of these four documented the tool used for monitoring,^38^ three documented the interval of observations^38^,^40^ and two described how the daily KMC duration was calculated.^38 39^

The three publications that combined caregiver and healthcare worker report documented the tool used to measure KMC duration,^29 31 32^ but only two documented the interval of observations, and none explained how the daily duration was computed.^29 32^ The publication that used a combination of an electronic device, healthcare worker report and caregiver report documented the interval of monitoring KMC duration but did not document the tool used or how the daily duration was computed.^30^ The publications that used independent observers documented the interval of observations and described how the daily KMC duration was computed.^33^,^35^

### Validation of the measurement methods

None of the publications with primary KMC duration data validated the method used to measure KMC duration. Only one publication measured the accuracy of a new device used to monitor skin-to-skin contact compared with healthcare worker report and caregiver report.^30^ Direct observation by healthcare workers was used as the reference standard against which an electronic monitoring device was compared for the purposes of accuracy; however, no validation was conducted. A maternal report was used to test the reliability of the electronic device to capture the duration of KMC at home. Four additional publications verified the consistency of measurements but did not undertake validation of the methods used.^25 29 31 38^ Two of these studies only compared the agreement between the observation by the healthcare workers and the parents without comparing with the set gold standard,^29 31^ while the other two only used a second person to verify entries without calculation of the agreement.^25 38^

## Discussion

In this scoping review, we found 213 publications on KMC of which 54 (25%) documented a method for measuring duration. Only 20 (9%) publications provided a detailed description of the KMC duration measurement method, and none reported validity. Most studies with a detailed description used caregiver report (9, 45%) or healthcare worker report (4, 20%). No framework for categorisation of KMC duration measurement methods was identified, and there was a lack of justification for the choice of method used for individual publications.

The observation that most studies did not document methods used to assess KMC duration is in accord with a previous systematic review, which found that more than 85% of studies did not include data on observations of KMC practice and that 45% lacked a description of KMC initiation and stopping criteria.^41^ Similarly, a most recent review that generated evidence leading to a policy change by WHO found that 19% (5 out of 27) of the included studies did not report on the duration of KMC.^10^

The lack of reliable measurement for the intervention dose (KMC duration) is an impediment to interpreting the evidence when meta-analyses that combine studies with different KMC measurement methods are used.^42 43^ Hence, although it is plausible that higher KMC duration could improve neonatal health outcomes,^44 45^ the evidence remains incomplete without more rigorously validated methods for measuring the dose of KMC. This is seen by variations in the evidence generated by different reviews where the Cochrane review (2016) found KMC reduction in mortality was only when the daily duration was 20 hours or more,^6^ while another found significant benefit when the daily duration was at least 8 hours.^10^ There were no published studies on the validation of methods used to measure KMC duration. This calls for studies to validate KMC duration measurement methods against a gold standard (a reliable method for continuous monitoring of KMC) to enable accurate data on KMC duration as an exposure, compared with outcomes such as mortality and morbidity. Video recording has been used in skin-to-skin care studies mainly for short duration like heel pricks procedures where the camera focuses on the neonate’s face not the environment.^46 47^ This could be an alternative as a gold standard against which the commonly used methods in KMC studies could be validated. However, continuous video recording of KMC has ethical challenges like limitations of anonymity and recording other non-research-related private experiences of the participants which might cause reluctance of the ethical committees to allow its use.^48^ The use of artificial intelligence (AI) platforms like it has been tried in a drug adherence trial could be a best alternative for a gold standard to evaluate the commonly used KMC duration measurement methods.^49^ This trial used visual confirmation of ingestion of the drug by using the AI platform mobile app and the same can be used to confirm skin-to-skin contact in KMC studies.^49^

Although WHO recommends KMC duration of at least 8 hours a day,^11^ there is limited evidence on the minimum duration of KMC with beneficial clinical effects given that the evidence base used to draw the recommendation found insufficient data on KMC duration less than 8 hours.^10^ Therefore, standardised operational definitions could improve this evidence base.^41^ In addition, our framework could help in guiding the selection and refining of indicators in routine information systems for assessing KMC duration as a marker for the quality of KMC.^50^ Chan and others have proposed indicators in the KMC measurement framework to include the duration of skin-to-skin contact,^41 50^ and the proposed framework for KMC duration measurement in this review will be helpful for the measurement of this indicator.

## Strengths and limitations of the study

### Strengths

This scoping review had several strengths. It followed Joanna Briggs Institute guidelines and was pre-registered on the Open Science Framework, ensuring transparency and methodological rigour. Two independent reviewers were involved, with a third resolving any disagreements, which minimised bias during study selection and data charting. The search strategy was comprehensive, covering both published and grey literature across multiple databases, with no restrictions on language or publication date. This helped reduce publication bias and expanded the scope of the review. Additionally, support from a clinical research specialist and pilot testing ensured accurate data collection and consistent analysis.

### Limitations

The review had several limitations and potential biases. By focusing solely on KMC duration and monitoring, we might have excluded studies on related practices, such as short procedural skin-to-skin care, potentially narrowing the scope and limiting relevant insights. Although the search had no language restrictions, five studies were excluded due to unavailable translations, which could reduce the comprehensiveness of the findings. Additionally, we did not review supplementary materials from included studies might have resulted in the omission of critical information, introducing bias in data interpretation and affecting the overall robustness of the review. These factors may have led to selective inclusion and gaps in the evidence base.

## Conclusion

KMC is a high-impact intervention for the survival of LBW neonates, but there is limited rigorous evaluation of the duration required. Reliable data on the dose response of KMC depends on the reliability of assessing its duration. This scoping review found most studies of KMC duration (91%) did not describe the methods used, and those that did were mainly reliant on caregiver report or healthcare worker report, both of which have limitations. Clarity is needed in reporting KMC duration measurement methods to increase comparability and rigour, and a validation study of gold standard versus caregiver report and healthcare worker report would be of value.

## supplementary material

10.1136/bmjopen-2023-079579 online supplemental file 1

10.1136/bmjopen-2023-079579 online supplemental file 2

## Data Availability

Data are available upon reasonable request.
