# Supplementary material for: Measuring the duration of kangaroo mother care for neonates: a scoping review
Source: BMJ Open. 2025 Jan 22;15(1):e079579. doi: 10.1136/bmjopen-2023-079579 (PMC11758684; doi:10.1136/bmjopen-2023-079579)
Supplement: online supplemental file 1 [file bmjopen-15-1-s001.pdf]

**Appendix 1: Search strategies**

| | **Ovid MEDLINE(R)**<br><br>**Date: 22/11/2022** | **Results per line** |
|---|---|---|
| 1 | infant, low birth weight/ or infant, small for gestational age/ or infant, very low birth weight/ or infant, extremely low birth weight/ or infant, premature/ or infant, extremely premature/ | 89966 |
| 2 | (low birth weight or small for gestational age or small gestational age or small for date infant$ or low birth weight or premature infant$ or premature neonate$ or prematurity or preterm infant$ or preterm neonate$ or premature babies or preterm babies or premature baby or preterm baby or premature newborn$ or preterm newborn$).mp. | 123783 |
| 3 | Premature Birth/ | 19559 |
| 4 | (preterm birth$ or premature birth$).mp. | 35716 |
| 5 | shorter gestation.mp. | 229 |
| 6 | preterm pregnancy.mp. | 132 |
| 7 | (sga or lbw or vlbw).mp. | 18124 |
| 8 | or/1-7 | 164261 |
| 9 | Kangaroo-Mother Care Method/ | 688 |
| 10 | (kangaroo mother care or kangaroo care).mp. | 1265 |
| 11 | skin to skin.mp. | 7174 |
| 12 | or/9-11 | 7943 |
| 13 | 8 and 12 | 1106 |
| 14 | ((monitor$ or measur$ or adher$) adj10 (kangaroo mother care or kangaroo care or "skin to skin")).mp. | 374 |
| 15 | (duration adj10 (kangaroo mother care or kangaroo care or "skin to skin")).mp. | 151 |
| 16 | or/13-15 | 1549 |

| | **Embase**<br><br>**Date: 22/11/2022** | **Results per line** |
|---|---|---|
| 1 | low birth weight/ or small for date infant/ | 56972 |
| 2 | very low birth weight/ or extremely low birth weight/ | 17072 |
| 3 | (low birth weight or small for gestational age or small gestational age or small for date infant$ or low birth weight or premature infant$ or premature neonate$ or prematurity or preterm infant$ or preterm neonate$ or premature babies or preterm babies or premature baby or preterm baby or<br><br>premature newborn$ or preterm newborn$).mp. | 202428 |
| 4 | prematurity/ | 119803 |
| 5 | (preterm birth$ or premature birth$).mp. | 41903 |
| 6 | shorter gestation.mp. | 289 |
| 7 | preterm pregnancy.mp. | 204 |
| 8 | (sga or lbw or vlbw).mp. | 27042 |
| 9 | or/1-8 | 225402 |
| 10 | kangaroo care/ | 1724 |
| 11 | (kangaroo mother care or kangaroo care).mp. | 2070 |
| 12 | skin to skin.mp. | 10333 |
| 13 | or/10-12 | 11652 |
| 14 | 9 and 13 | 1594 |
| 15 | ((monitor$ or measur$ or adher$) adj10 (kangaroo mother care or kangaroo care or "skin to skin")).mp. | 540 |
| 16 | (duration adj10 (kangaroo mother care or kangaroo care or "skin to skin")).mp. | 216 |
| 17 | or/14-16 | 2217 |

| | **APA PsycInfo**<br><br>**Date: 22/11/2022** | **Results per line** |
|---|---|---|
| 1 | Birth Weight/ | 3453 |
| 2 | (low birth weight or small for gestational age or small gestational age or small for date infant$ or low birth weight or premature infant$ or premature neonate$ or prematurity or preterm infant$ or preterm neonate$ or premature babies or preterm babies or premature baby or preterm baby or<br><br>premature newborn$ or preterm newborn$).mp. | 10046 |
| 3 | premature birth/ | 6254 |
| 4 | (preterm birth$ or premature birth$).mp. | 7931 |
| 5 | shorter gestation.mp. | 42 |
| 6 | preterm pregnancy.mp. | 2 |
| 7 | (sga or lbw or vlbw).mp. | 2008 |
| 8 | or/1-7 | 13930 |
| 9 | (kangaroo mother care or kangaroo care).mp. | 183 |
| 10 | skin to skin.mp. | 519 |
| 11 | or/9-10 | 615 |
| 12 | 8 and 11 | 187 |
| 13 | ((monitor$ or measur$ or adher$) adj10 (kangaroo mother care or kangaroo care or "skin to skin")).mp. | 56 |
| 14 | (duration adj10 (kangaroo mother care or kangaroo care or "skin to skin")).mp. | 21 |
| 15 | or/12-14 | 249 |

| | **Cochrane Central Register of Controlled Trials (CENTRAL) and Cochrane Database of Systematic Reviews (CDSR)**<br><br>**Date: 22/11/2022** | **Results per line** |
|---|---|---|
| #1 | MeSH descriptor: [Infant, Low Birth Weight] explode all trees | 2338 |
| #2 | MeSH descriptor: [Infant, Premature] explode all trees | 4277 |
| #3 | “low birth weight” or “small for gestational age” or “small gestational age” or “small for date” NEXT infant* or “low birth weight” or premature NEXT infant* or premature NEXT<br><br>neonate* or prematurity or preterm NEXT infant* or preterm NEXT neonate* or “premature babies” or “preterm babies” or “premature baby” or “preterm baby” or premature NEXT newborn* or preterm NEXT newborn* | 19540 |
| #4 | MeSH descriptor: [Premature Birth] this term only | 1814 |
| #5 | preterm NEXT birth* or premature NEXT birth* | 4614 |
| #6 | "shorter gestation" | 11 |
| #7 | "preterm pregnancy" | 26 |
| #8 | sga or lbw or vlbw | 3218 |
| #9 | #1 or #2 or #3 or #4 or #5 or #6 or #7 or #8 | 22856 |
| #10 | MeSH descriptor: [Kangaroo-Mother Care Method] this term only | 111 |
| #11 | "kangaroo mother care" or "kangaroo care" | 651 |
| #12 | "skin to skin contact" or "skin to skin care" | 547 |
| #13 | #10 or #11 or #12 | 1002 |

| | Clinicaltrials.gov | Results |
|---|---|---|
| | **Date: 18/11/2022** | |
| | kangaroo OR "skin to skin" | 119 |

| | WHO International Clinical Trials Registry Platform (ICTRP) | Results |
|---|---|---|
| | **Date: 18/11/2022** | |
| | kangaroo | 203 |

| | The ISRCTN registry | Results |
|---|---|---|
| | **Date: 21/11/2022** | |
| | Kangaroo https://www.isrctn.com/search?q=kangaroo+ | 12 |

| | Latin American and Caribbean Health Sciences Literature (LILACS) | Results |
|---|---|---|
| | **Date: 21/11/2022** | |
| | Kangaroo | 315 |

| | MedRxiv | Results |
|---|---|---|
| | **Date: 21/11/2022** | |
| | “skin to skin” | 166 |

| | **African Index Medicus** | **Results** |
|---|---|---|
| | **Date: 21/11/2022** | |
| | Kangaroo | 9 |

| | **Open Grey libraries, references from relevant systematic reviews and websites of the Kangaroo Foundation** | **Results** |
|---|---|---|
| | **Date: 21/11/2022** | |
| | **Open Grey libraries:**<br>search term “skin to skin”<br>AND<br>Search terms Kangaroo care or kangaroo unit*<br><br>**References and google scholar:**<br>"kangaroo mother care" or "kangaroo care"<br>OR<br>“skin to skin contact" or "skin to skin care" | 175 |
