# Supplementary material for: Measuring the duration of kangaroo mother care for neonates: a scoping review
Source: BMJ Open. 2025 Jan 22;15(1):e079579. doi: 10.1136/bmjopen-2023-079579 (PMC11758684; doi:10.1136/bmjopen-2023-079579)
Supplement: online supplemental file 2 [file bmjopen-15-1-s002.pdf]

Table 1. Publications with a detailed description of KMC duration measurement

First author, year	Publication title	Study design & sample size	Setting: country; level of NICU	Method for KMC duration	Method details	Comments
M. J. F. Goudard; 2022	Skin-to-skin contact and deaths in new-borns weighing up to 1800 grams: a cohort study	Observational study; 405	Brazil	Caregiver & Healthcare worker	SSC time was recorded on cards by the health team at the beginning then, parents performed the registration, under the supervision of healthcare workers.	☑ Mention of data collection tool used to record KMC duration.<br>☑ Interval of observation documented.<br>☐ Calculation of daily KMC duration documented
S. Jegannathan; 2022	Quality improvement initiative to improve the duration of Kangaroo Mother Care in tertiary care neonatal unit of South India	Observational study; 86	India	Caregiver report (KMC charts)	KMC Charts were given to KMC mothers to mark daily hours of KMC by mothers.	☑ Mention of data collection tool used to record KMC duration.<br>☐ Interval of observation documented.<br>☐ Calculation of daily KMC duration documented
A. Joshi; 2022	Quality improvement in Kangaroo Mother Care: learning from a teaching hospital	Observational study; 86	India: NICU level unknown	Caregiver report (self-report)	The nurse noted the previous day's, KMC hours and other information as reported by the mother.	☐ Mention of data collection tool used to record KMC duration.<br>☐ Interval of observation documented.<br>☐ Calculation of daily KMC duration documented.
Nahya Salim; 2021	Kangaroo mother care: EN-BIRTH multi-country validation study	Observational study; 840	Tanzania, Napal & Bangladesh	Independent observer	Observers monitored components of KMC hourly in some settings and 12-hourly in other settings	☑ Mention of data collection tool used to record KMC duration.<br>☑ Interval of observation documented.<br>☑ Calculation of daily KMC duration documented.

Detailed methodological description of; 1) tools used to document KMC duration monitoring, 2) the interval of the observations and, 3) how the total or daily KMC duration was calculated from the observations ☑= Yes ☐=No.

KMC=Kangaroo Mother Care, KC= Kangaroo Care, NICU= Neonatal Intensive Care Unit, EN-BIRTH= Every Newborn Birth Indicators Research Tracking in Hospitals, STS=Skin to Skin, SSC=Skin-to-skin care, HCW=Health care worker, CG=Caregiver

First author, year	Publication title	Study design & sample size	Setting: country; level of NICU	Method for KMC duration	Method details	Comments
E. A. Adejuyigbe; 2021	Impact of continuous Kangaroo Mother Care initiated immediately after birth on survival of newborns with birth weight between 1.0 to < 1.8 kg	RCT; 4200	Ghana, Tanzania, Malawi, Nigeria & India	Independent observer	Information on the duration of SSC contact and the duration of hospital stay was collected by research assistants	☐ Mention of data collection tool used to record KMC duration.<br>☐ Interval of observation documented.<br>☐ Calculation of daily KMC duration documented.
H. Brotherton; 2021	Impact of early kangaroo mother care versus standard care on survival of mild-moderately unstable new-borns <2000 grams	RCT; 279	The Gambia: NICU level not specified	Healthcare worker report	Research nurses observed and recorded KMC duration and position. Documented timing of each KMC session, KMC provider and reason for coming out of KMC position.	☐ Mention of data collection tool used to record KMC duration.<br>☐ Interval of observation documented.<br>☐ Calculation of daily KMC duration documented
K. Chavula; 2020	Improving Skin-to-Skin Practice for new-borns in Kangaroo Mother Care in Malawi through the use of a customized baby wrap: A randomized control trial	RCT; 301	Malawi; NICU level II	Caregiver report (self-report)	Mothers reported practicing SSC post-discharge & duration more than half the day and more than half the night post-discharge.	☐ Mention of data collection tool used to record KMC duration.<br>☐ Interval of observation documented.<br>☐ Calculation of daily KMC duration documented
C. Sahlen Helmer; 2020	A Randomized Trial of Continuous Versus Intermittent Skin-to-Skin Contact After Premature Birth and the Effects on Mother-Infant Interaction	RCT; 31	Sweden; NICU level unknown	Caregiver report (KMC charts)	Parents in continuous SSC documented who provided SSC and whether they were off SSC for any reason. Parents in the intermittent group registered when and for how long they provided SSC.	☑ Mention of data collection tool used to record KMC duration.<br>☑ Interval of observation documented.<br>☐ Calculation of daily KMC duration documented.

Detailed methodological description of; 1) tools used to document KMC duration monitoring, 2) the interval of the observations and, 3) how the total or daily KMC duration was calculated from the observations ☑= Yes ☐=No.

KMC=Kangaroo Mother Care, KC= Kangaroo Care, NICU= Neonatal Intensive Care Unit, EN-BIRTH= Every Newborn Birth Indicators Research Tracking in Hospitals, STS=Skin to Skin, SSC=Skin-to-skin care, HCW=Health care worker, CG=Caregiver

First author, year	Publication title	Study design & sample size	Setting: country; level of NICU	Method for KMC duration	Method details	Comments
B.S. Tandberg; 2018	Parent-Infant Closeness, Parents' Participation, and Nursing Support in Single-Family Room and Open Bay NICUs	Observational; 64	Norway: NICU level unknown	Caregiver report (KMC chart)	Parents recorded the duration of SSC care in a KMC diary. Diary entries were made daily for the first 14 days following inclusion in the study. Parents reported hour by hour during these 14 days.	☑ Mention of data collection tool used to record KMC duration.<br>☑ Interval of observation documented.<br>☐ Calculation of daily KMC duration documented
S. Rao; 2018	Continuous, real-time monitoring of neonatal position and temperature during Kangaroo Mother Care using a wearable sensor: a techno-feasibility pilot study	Observational; 12	India: NICU level III	Electronic monitoring device, healthcare worker's direct observation & Maternal self-report	Baby's position captured as presence of "touch" between the device and the skin of baby/mother was compared against reported/ observed KMC episodes". In the hospital, the research nurse annotated the starting and ending times of KMC by direct observation. At home KMC duration was self-reported by the mother. Direct observation was the "reference standard" in the hospital against which the device was compared for purposes of validation.	☐ Mention of data collection tool used to record KMC duration.<br>☐ Interval of observation documented.<br>☐ Calculation of daily KMC duration documented

Detailed methodological description of; 1) tools used to document KMC duration monitoring, 2) the interval of the observations and, 3) how the total or daily KMC duration was calculated from the observations ☑= Yes ☐=No.

KMC=Kangaroo Mother Care, KC= Kangaroo Care, NICU= Neonatal Intensive Care Unit, EN-BIRTH= Every Newborn Birth Indicators Research Tracking in Hospitals, STS=Skin to Skin, SSC=Skin-to-skin care, HCW=Health care worker, CG=Caregiver

First author, year	Publication title	Study design & sample size	Setting: country; level of NICU	Method for KMC duration	Method details	Comments
H.C. Watkins; 2018	Observation study showed that the continuity of skin-to-skin contact with low-birthweight infants in Uganda was suboptimal	Observational; 12	Uganda: NICU level II	Independent observer	Continuous observation of infants began after birth and up to day 7 of life, discharge, or death, whichever came first. The total duration of hours spent in SSC was calculated by adding together the duration of all individual SSC sessions on that day. If an infant received at least 20 hours of SSC, it was considered continuous KMC, and any fewer hours of SSC per day was documented as intermittent KMC.	☐ Mention of data collection tool used to record KMC duration.<br>☐ Interval of observation documented.<br>☑ Calculation of daily KMC duration documented
P. Oras; 2016	Skin-to-skin contact is associated with earlier breastfeeding attainment in preterm infants	Observational; 104	Sweden: NICU level III	Caregiver report & healthcare worker	Skin to skin duration was recorded by the parents or by staff on a detailed form. Median daily SSC duration was the data used for the study.	☑ Mention of data collection tool used to record KMC duration.<br>☐ Interval of observation documented.<br>☐ Calculation of daily KMC duration documented
A. Soni; 2016	The presence of physician champions improved Kangaroo Mother Care in rural western India	Observational; 648	India: NICU level II	Healthcare worker report	Nursing staff documented KMC duration using a standardized form &<br>recorded information for eight days. The charts were incorporated into the neonate's medical notice updated daily by the nurses and reviewed daily by the physician.	☑ Mention of data collection tool used to record KMC duration.<br>☑ Interval of observation documented.<br>☑ Calculation of daily KMC duration documented.

Detailed methodological description of; 1) tools used to document KMC duration monitoring, 2) the interval of the observations and, 3) how the total or daily KMC duration was calculated from the observations ☑= Yes ☐=No.

KMC=Kangaroo Mother Care, KC= Kangaroo Care, NICU= Neonatal Intensive Care Unit, EN-BIRTH= Every Newborn Birth Indicators Research Tracking in Hospitals, STS=Skin to Skin, SSC=Skin-to-skin care, HCW=Health care worker, CG=Caregiver

First author, year	Publication title	Study design & sample size	Setting: country; level of NICU	Method for KMC duration	Method details	Comments
J. Pervin; 2015	Implementing Kangaroo mother care in a resource-limited setting in rural Bangladesh	Observational; 423	Bangladesh: NICU level I	Healthcare worker report	Nursing attendants noted the beginning and end of every skin-to-skin contact session and calculated skin-to-skin contact time for each session. The times of each session in the 24h period was added together to determine total skin-to-skin contact duration per day.	☐ Mention of data collection tool used to record KMC duration.<br>☑ Interval of observation documented.<br>☑ Calculation of daily KMC duration documented
J. Gonya; 2013	Factors associated with maternal visitation and participation in skin-to-skin care in an all-referral level IIIc NICU	Observational; 32	USA: NICU level III	Caregiver report (KMC chart)	A log was provided to mothers who recorded when they visited the small baby NICU, if they participated in SSC, how long they participated in SSC, and if there were any issues involved in the SSC process.	☑ Mention of data collection tool used to record KMC duration.<br>☑ Interval of observation documented.<br>☐ Calculation of daily KMC duration documented
Y.T. Blomqvist; 2013	Initiation and extent of skin-to-skin care at two Swedish neonatal intensive care units	Observational; 104	Sweden: NICU level III	Caregiver report & healthcare worker	Time of initiation of SSC and who provided this care were recorded continuously in the infants' medical charts by either the parents or the NICU staff. The reliability of parents' registrations of the time spent with SSC had been assessed prior to the study.	☑ Mention of data collection tool used to record KMC duration.<br>☑ Interval of observation documented.<br>☐ Calculation of daily KMC duration documented.

Detailed methodological description of; 1) tools used to document KMC duration monitoring, 2) the interval of the observations and, 3) how the total or daily KMC duration was calculated from the observations ☑= Yes ☐=No.

KMC=Kangaroo Mother Care, KC= Kangaroo Care, NICU= Neonatal Intensive Care Unit, EN-BIRTH= Every Newborn Birth Indicators Research Tracking in Hospitals, STS=Skin to Skin, SSC=Skin-to-skin care, HCW=Health care worker, CG=Caregiver

First author, year	Publication title	Study design & sample size	Setting: country; level of NICU	Method for KMC duration	Method details	Comments
R. Flacking; 2013	Influence of NICU co-care facilities and skin-to-skin contact on maternal stress in mothers of preterm infants	Mixed methods feasibility study; 300	Sweden: NICU level III	Caregiver report (KMC chart)	Caregivers provided KMC duration self-reports using calendars. Parents marked the initiation and ending of each SSC episode. At the end of the 2-week period, the nurse revisited the mother, collected the calendar, and provided a new calendar for the following 2-week period.	☑ Mention of data collection tool used to record KMC duration. ☑ Interval of observation documented. ☐ Calculation of daily KMC duration documented
R. Flacking; 2011	Positive Effect of Kangaroo Mother Care on Long-Term Breastfeeding in Very Preterm Infants	Observational; 300	Sweden: NICU level unknown	Caregiver report (KMC chart)	KMC data gathered through self-reports by caregivers in the format of calendars. Parents marked the initiation and ending of each skin-to-skin episode rounded to the nearest 5- or 10-minute interval.	☑ Mention of data collection tool used to record KMC duration. ☑ Interval of observation documented. ☐ Calculation of daily KMC duration documented.
G.C. Anderson; 2003	Mother-Newborn Contact in a Randomized Trial of Kangaroo (Skin-to-Skin) Care	RCT; 91	USA: NICU level unknown	Caregiver report (KMC chart)	Contact logs used to document KMC duration (when the contact began and ended) & by whom. Caregivers completed the contact log and researcher verified each entry with the mothers at the end of each 8-hour shift.	☑ Mention of data collection tool used to record KMC duration. ☑ Interval of observation documented. ☐ Calculation of daily KMC duration documented
R. Feldman; 2003	Skin-to-skin contact (Kangaroo Care) accelerates autonomic and neuro-behavioural maturation in preterm infants	Observational; 70	Israel: NICU level unknown	Healthcare worker report	During KC infants were observed by the nurses who recorded the exact times when the mothers and infants remained in skin-to-skin contact and when the infant returned to standard incubator care.	☐ Mention of data collection tool used to record KMC duration. ☑ Interval of observation documented. ☐ Calculation of daily KMC duration documented.

Detailed methodological description of; 1) tools used to document KMC duration monitoring, 2) the interval of the observations and, 3) how the total or daily KMC duration was calculated from the observations ☑= Yes ☐=No.

KMC=Kangaroo Mother Care, KC= Kangaroo Care, NICU= Neonatal Intensive Care Unit, EN-BIRTH= Every Newborn Birth Indicators Research Tracking in Hospitals, STS=Skin to Skin, SSC=Skin-to-skin care, HCW=Health care worker, CG=Caregiver
